# Patchiness and Co-Existence of Indigenous and Invasive Mussels at Small Spatial Scales: The Interaction of Facilitation and Competition

**DOI:** 10.1371/journal.pone.0026958

**Published:** 2011-11-22

**Authors:** Johan Erlandsson, Christopher D. McQuaid, Martin Sköld

**Affiliations:** 1 ARONIA Coastal Zone Research Team, Åbo Akademi University/Novia University of Applied Sciences, Ekenäs, Finland; 2 Coastal Research Group, Department of Zoology and Entomology, Rhodes University, Grahamstown, South Africa; 3 Department of Mathematics, Stockholm University, Stockholm, Sweden; National Institute of Water & Atmospheric Research, New Zealand

## Abstract

Ecological theory predicts that two species with similar requirements will fail to show long-term co-existence in situations where shared resources are limiting, especially at spatial scales that are small relative to the size of the organisms. Two species of intertidal mussels, the indigenous *Perna perna* and the invasive *Mytilus galloprovincialis*, form mixed beds on the south coast of South Africa in a situation that has been stable for several generations of these species, even though these populations are often limited by the availability of space. We examined the spatial structure of these species where they co-exist at small spatial scales in the absence of apparent environmental heterogeneity at two sites, testing: whether conspecific aggregation of mussels can occur (using spatial Monte-Carlo tests); the degree of patchiness (using Korcak B patchiness exponent), and whether there was a relationship between percent cover and patchiness. We found that under certain circumstances there is non-random conspecific aggregation, but that in other circumstances there may be random distribution (i.e. the two species are mixed), so that spatial patterns are context-dependent. The relative cover of the species differed between sites, and within each site, the species with higher cover showed low Korcak B values (indicating low patchiness, i.e. the existence of fewer, larger patches), while the less abundant species showed the reverse, i.e. high patchiness. This relationship did not hold for either species within sites. We conclude that co-existence between these mussels is possible, even at small spatial scales because each species is an ecological engineer and, while they have been shown to compete for space, this is preceded by initial facilitation. We suggest that a patchy pattern of co-existence is possible because of a balance between direct (competitive) and indirect (facilitative) interactions.

## Introduction

General ecological theory predicts that stable coexistence of two or more similar species will be unusual and dependent on resource availability or predation [1], [2], [3]. Coexistence of competing species at larger local scales, that is scales beyond those at which species directly interact, is often explained by environmental heterogeneity, or by species interactions generating spatially segregated distributions even in the absence of environmental heterogeneity [1], [3], [4], [5], [6], [7]. Thus, at spatial scales that are small relative to body size, it is considered unlikely that two competing species can coexist, unless the inferior competitor is a superior colonizer of empty patches or there is spatial variability in their competitive abilities so that the superior competitor is at a disadvantage in at least some part of its distribution [2], [6], [8]. It is even less likely that two related species are able to coexist if one is an introduced species and the other indigenous, since the introduced species is often capable of becoming invasive precisely because it is a superior competitor [2].

Exploitative competition results in indirect negative interactions, while interference competition involves direct negative interactions, e.g. territoriality, overgrowth or chemical competition. In classical competition theory, there are no benefits, only costs, for species engaged in interference competition. In nature, however, interference between interacting species can be both costly and beneficial for an inferior resource exploiter, e.g. the production of chemicals as defence involves a cost, while predation on the eggs and larvae of the competitor is beneficial in terms of growth etc. [2].

Competition will occur only if the resource is limiting, and resources can be either renewable or non-renewable. In the case of exploitation competition for a non-renewable resource, this can lead to systems being controlled by lotteries, the classic example being coral reef fishes competing for habitat [9]. In benthic marine systems competition is primarily for either food, which is renewable, or for space, especially primary space, which is often non-renewable [10]. Competition for space in these systems involves both exploitation, with some species being faster at colonising space than others [11], and interference competition with overgrowth being a common mode of interaction [12], [13]. Because space is an absolute requirement for sessile or sedentary species, it is more likely to lead to competitive exclusion [10]. It has been suggested that introduced alien species are more likely to be successful if they are dissimilar from the dominant indigenous species, for example if they belong to different genera [14], [15], precisely because they will have less similar resource requirements. However, in the case of competition for primary space (as opposed to secondary space) it is difficult to see how the resource can be differentiated and it is unclear that taxonomic similarity will have a strong effect on invasive success. For example, the introduced Mediterranean mussel *Mytilus galloprovincialis* (*Lamarck 1819*) has replaced the indigenous mussel *Aulacomya ater* (Chemn.) on the west coast of South Africa [16] but competes for space equally aggressively with congeneric *M. trossulus* (*Gould 1850*), on the west coast of the USA, excluding it from the southern end of its distribution [17], [18].

In South Africa *M. galloprovincialis* now poses a threat on the south coast to the indigenous mussel *Perna perna* (*Linnaeus 1758*) [19], which has generally lower fecundity, recruitment and growth rates than other mytilid mussels [20], [21]. At present, the abundance of *M. galloprovincialis* on the south coast is highly variable and site-specific [22]. At sites where *M. galloprovincialis* is abundant, it dominates the upper mussel zone, and *P. perna* the lower zone, with an overlap in distribution (co-existence at cm-m scales) in the mid zone [19]. Being different species, these mussels have different tolerances to wave action and desiccation, and in the upper zone they exhibit different post-settlement mortality of recent recruits, factors that partially explain these patterns of zonation [23], [24], [25], [26]. Thus, the two species exhibit partial habitat partitioning across a gradient of environmental conditions, exhibited as differences in zonation. Although habitat partitioning through differences in physiological tolerances can be interpreted as a consequence of past competition, this does not apply to this situation where one of the species has been recently introduced to the system, rather habitat segregation reflects both direct and indirect interactions between the two. Direct interference competition between *P. perna* and *M. galloprovincialis* occurs through different growth rates, so that they can usurp each other's space [24], [27]. Exploitative competition between mussels includes different settlement and recruitment intensity [25] that results in different recolonization rates after disturbance. *M. galloprovincialis* re-colonizes free space faster than *P. perna* in the sympatric mid zone after disturbance by severe storm waves [11]. This accords with the suggestion of Amarasekare [2] that disturbance or human exploitation can help invasive species to dominate, at least temporarily.

In addition to competition for space, there is indirect evidence that these mussels are likely to compete for food. Depletion of food in the water column seems unlikely, but mussel growth rates can vary in response to very small scale (10 s cm) changes in food availability [28], while van de Koppel et al. [3] have shown that self-organized spatial structure in *M. edulis* is driven partly by food depletion, and Bertness and Grosholz [29] identify one of the disadvantages for bivalves of living in aggregations as reduced growth through intraspecific competition for food. As we can imagine no mechanism for partitioning food between our two species, we assume they exhibit interspecific competition for this resource. Certainly, they have indistinguishable stable isotope (δC^13^ and δ^15^) signatures [30], while upwelling, which increases local food availability, strongly affects their abundances and size distribution [31].

Although *M. galloprovincialis* and *P. perna* exhibit partial habitat segregation, and have similar requirements for food and space, they also exhibit co-existence in the mid-mussel zone that seems to be stable in the mid-term i.e. over decades or multiple generations [19]. Facilitation or positive interactions between species [32] is believed to be less common between closely related invasive and indigenous species than between taxonomically distinct organisms [14], yet *P. perna* and *M. galloprovincialis* exhibit not just competitive interactions, they also facilitate each other's survival in at least three ways. This is essentially because they are both ecosystem engineers so that each species can moderate abiotic conditions enough to allow survival of the other in areas where the second species would otherwise be excluded. Firstly, *P. perna* rarely settles on bare rock and its recruitment rates are facilitated by the much faster recolonisation of bare rock surfaces of *M. galloprovincialis* in the mid zone following disturbances such as storms [11]. Secondly, *M. galloprovincialis* survives wave action much better on the low shore when it is intermingled experimentally with *P. perna* than in monospecific plots [23], [24]. Thirdly, *P. perna* survives desiccation in the mid-mussel zone better when mingled experimentally with the more tolerant *M. galloprovincialis* [27]. Thus, both competition and facilitation occur between these two mussel species.

Consequently, this system allows us to examine co-existence at small spatial scales between two species competing for the same resources (food and space) in the absence of apparent environmental heterogeneity. If interspecific competition is of over-riding importance, we would expect some degree of conspecific aggregation due to small-scale competitive exclusion. However, because the two species can facilitate each other's survival on parts of the shore where they are otherwise vulnerable to either desiccation (*P. perna*) or wave action (*M. galloprovincialis*), we anticipate that individuals of each species would be likely to exist close to individuals of the other species, i.e. being more mixed (randomly dispersed), in the mid zone. Here we examine the small-scale spatial structure (at cm scales) of these two mussels where they co-exist in the mid-mussel zone. In particular, we examine the link between abundance (percent cover) and the degree of patchiness of each species to estimate whether numerical dominance of a species is an important predictor of patchiness, and also ask whether mussels in mixed beds are significantly aggregated with conspecifics.

## Methods

### Study area and sampling design

We studied two sites where *M. galloprovincialis* occurs at high densities on the south coast of South Africa [22]. The sites, Look-Out Beach and Robberg, are rocky shores separated by ca 5 km in Plettenberg Bay (34°05′S; 23°20′E). Intertidal mussel beds in this region are wave exposed and can be divided into low, mid- and upper mussel zones. The width of each mussel zone depends on the slope of the shore and the degree of wave action, but generally each zone is 2–3 metres wide. Mussel beds are usually monolayered rather than multilayered, with the byssus threads of adults attached directly to the rock surface [33]. The two species are interspersed in the mid-mussel zone to form a mosaic within patches of 100% cover, although percent cover in the overall zone may be <100% as patches can be separated by unoccupied space.

Two sub-sites, separated by ca 25 m, were sampled at each site during consecutive spring low tides. Within each sub-site, non-overlapping photographs (real surface area 30×20 cm^2^) were taken haphazardly within a 5 m stretch in the mid-mussel zone. We took ca 30 photographs per sub site to ensure sufficient good quality images and analysed a sub-set of these. In each of 10 randomly selected photographs, the Korcak B patchiness exponent was estimated for the small-scale distribution of one of the two mussel species (*M. galloprovincialis* or *P. perna*) and 10 other photos were selected for the other species so that the degree of patchiness for each species could be estimated independently (see below). Using 3 photographs selected randomly from the 30 taken at one of the sub-sites from each site, we tested the hypothesis of non-random small-scale aggregation, with the null hypothesis being random distribution (see below). Discrimination between the two mussel species in the photographs was based mainly on shell colour, *M. galloprovincialis* having darker and blue colours (sometimes black) and *P. perna* having brown (usually light brown) and more purple colours [34]. Where there was uncertainty, we used more subtle shell colour differences (e.g. narrow blue lines indicate *M. galloprovincialis*, while reddish tints characterise *P. perna*) described by Bownes [35] and Bownes et al. [36]. In addition, the percent cover of each mussel species was estimated in a 10×10 cm^2^ quadrat in each photograph to examine the relationship between cover and patchiness. Cover was estimated visually using a transparent frame of 16 small quadrats (each constituting 6.25%) put over the larger quadrat within each photo.

### Analyses and modelling

Different methods and techniques of estimating spatial heterogeneity and patchiness exist, including geostatistical techniques such as spatial autocorrelation, variograms/semivariograms and fractal analysis/dimension, that have also been used in intertidal systems [37], [38], [39], [40]. However, in the present study we needed an index that would measure patch sizes of each species directly while also estimating the size distribution of patches over different continuous small scales (in contrast to e.g. variance/mean ratio). The Korcak B patchiness exponent is such an index and therefore related to the concept of fractal dimension [41], [42]. B is an estimate of the slope of the logarithmic relationship between the number of patches ≥ specific threshold sizes and the different threshold sizes of those patches (here estimated as the number of individuals in a patch; see below). Thus, the number of patches ≥ the specific predetermined sizes is plotted on a log-linear graph [41], [42], [43]. The Korcak B patchiness exponent was originally used for describing the distribution/numbers of different sized islands (different areas) in an archipelago, but has since been used for different purposes although this has been rare in ecology [41]. It is also a useful index for the estimation of habitat fragmentation [42], [43] since it takes into account two of the three criteria for this process, i.e. the higher number of patches produced and the division into smaller patches [44]. The greater isolation/separation between patches observed during the habitat fragmentation process is not estimated by this technique as it is with, for example, the nearest-neighbour analysis, but the nearest-neighbour analysis does not estimate or take into account the higher number of smaller patches produced. Our second technique, Monte-Carlo testing, used to assess deviation from random patterns is, together with other random permutation tests, commonly used in ecological studies [45]. What is special about the technique used in the present study is the application of a spatially explicit Monte-Carlo technique in a user-friendly way.

#### Deviation from small-scale random distributions

**Figure 1:**
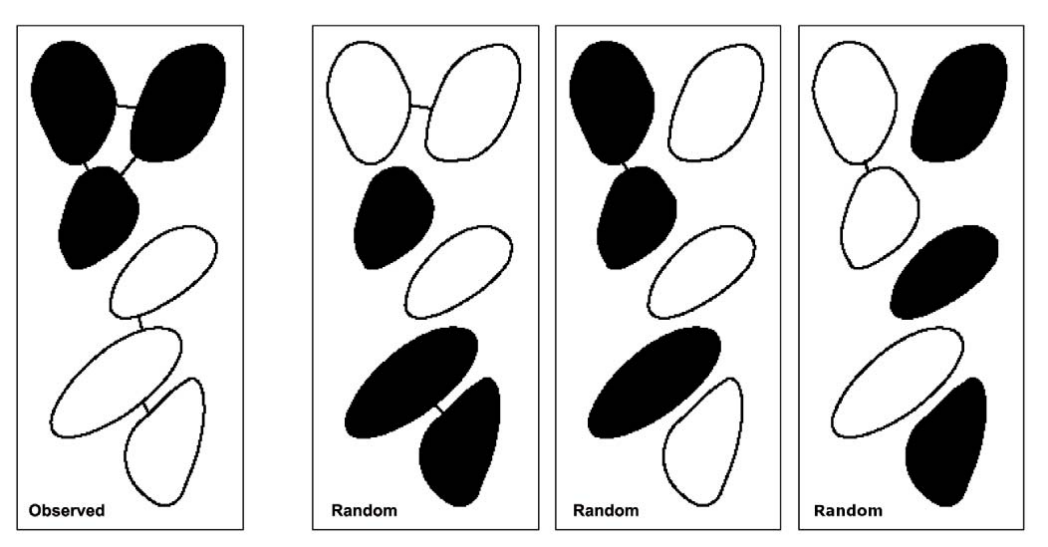
Simplified illustration of Monte-Carlo approach. Hypothetical configuration (observed; randomized) showing aggregations of two species (black/white). Neighbouring conspecifics are joined by arcs. Our measure of the degree of conspecific aggregation equals the number of arcs in the frame (i.e. 5 in frame 1). Frames 2-4: Sample random reconfigurations of the species labels, the number of arcs being 2, 1 and 1.

Here, our aim was to test the hypothesis that individuals of the two mussel species were non-randomly aggregated with their conspecifics at small scales in the 10×10 cm^2^ quadrats in each of the 3 photographs at one sub-site from each site. As a measure of the degree of conspecific aggregation, we counted the total number of pairs of neighbouring mussels that belonged to the same species within each quadrat of the photos (the total density of mussels in these 6 photos ranged from 40 to 122). We also took into account the mussels just outside each quadrat, so that all neighbours to mussels inside the quadrat were detected, in order to avoid edge effects. Two mussels were judged to form a neighbouring pair if they were connected by shell contact or separated by a distance equal to or less than half of the width of a mussel individual without their boundaries being intersected by another mussel. This allowed for possible shell contact when the shell valves were open and possible byssus contact. The tests were performed conditionally on the observed number of each species and their spatial configuration with species labels blinded. Under these circumstances, random samples of configurations can easily be generated by randomly assigning species to the mussels while keeping their relative numbers fixed (see Fig. 1). For each photo, a Monte-Carlo based p-value for the hypothesis under study was computed as the proportion of a large number of generated random species configurations with the degree of conspecific aggregation greater than the observed. For the Monte-Carlo tests, the sample unit was the individual mussel, so that sample size was the number of mussels in each quadrat. To allow a more robust test, we subsequently combined the probabilities derived from the 3 photographs for each site using Fisher's method [46], providing meta-analyses of the results.

#### Patchiness structure of aggregations

To describe the spatial structure of the aggregations (degree of patchiness) of the two mussel species in the mid zone, we used the Korcak patchiness exponent B [41]. In each of 10 photographs at each sub-site we counted the number of patches or clumps (N) with the number of mussel individuals (10 different photos for each species) larger or equal (≥) to i where i is equal to 1, 2, 4, 8, and 16 individuals. An aggregation was defined as mussels of each species that were connected by shell contact or a distance equal to or less than half of the width of a mussel individual between mussels. The Korcak patchiness exponent B is estimated from:
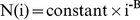
(1)or
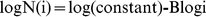
(2)B is estimated from the slope of the log-log relationship between N and i. Higher B estimates indicate smaller aggregations of individuals and more isolated mussels (i.e. higher patchiness), while lower B estimates mean that mussels are distributed in larger clumps with more individuals in each clump (i.e. lower patchiness).

Three-factor ANOVA (species as a fixed factor; site as a random factor; subsite as a random factor nested in site) was done for B aggregation estimates and for percent cover. Correlation analysis between percent cover and B of each species and site (and pooled data) was also conducted.

## Results

### Deviation from random distributions

The Monte-Carlo test of random distribution of species gave p-values of 0.00001, 0.013, and 0.11 respectively for the three photos at Look-out Beach and values of 0.013, 0.053 and 0.22 at Robberg. While not all of these p-values showed significant deviation (i.e. p<0.05) from randomness, they may be combined site-wise using Fisher's method [46], which gives a p-value of 0.0001 for Look-out Beach and 0.006 at Robberg. Hence, our test supports the interpretation that non-random aggregations can occur at both sites. However, this can be seen as a pilot study as we can neither accept nor reject the possibility that non-random aggregations exist over the whole spatial scale of these sites, since only a very small subset of this was examined. Thus, under certain circumstances there is non-random conspecific aggregation of *P. perna* and *M. galloprovincialis* (i.e. context-dependent pattern), but random distribution (i.e. the two species are mixed at small scales) also occurs, meaning that neither conspecific or random aggregation is the rule at these sites. Since we did not design the study to analyse the circumstances or contexts that promote either random or aggregated patterns, there was no reason to analyse further samples. Instead we focus on describing the degree of patchiness of each species separately at each site, and whether there was any relationship between patchiness and percent cover of each species.

### Patchiness structure of aggregations

**Figure 2:**
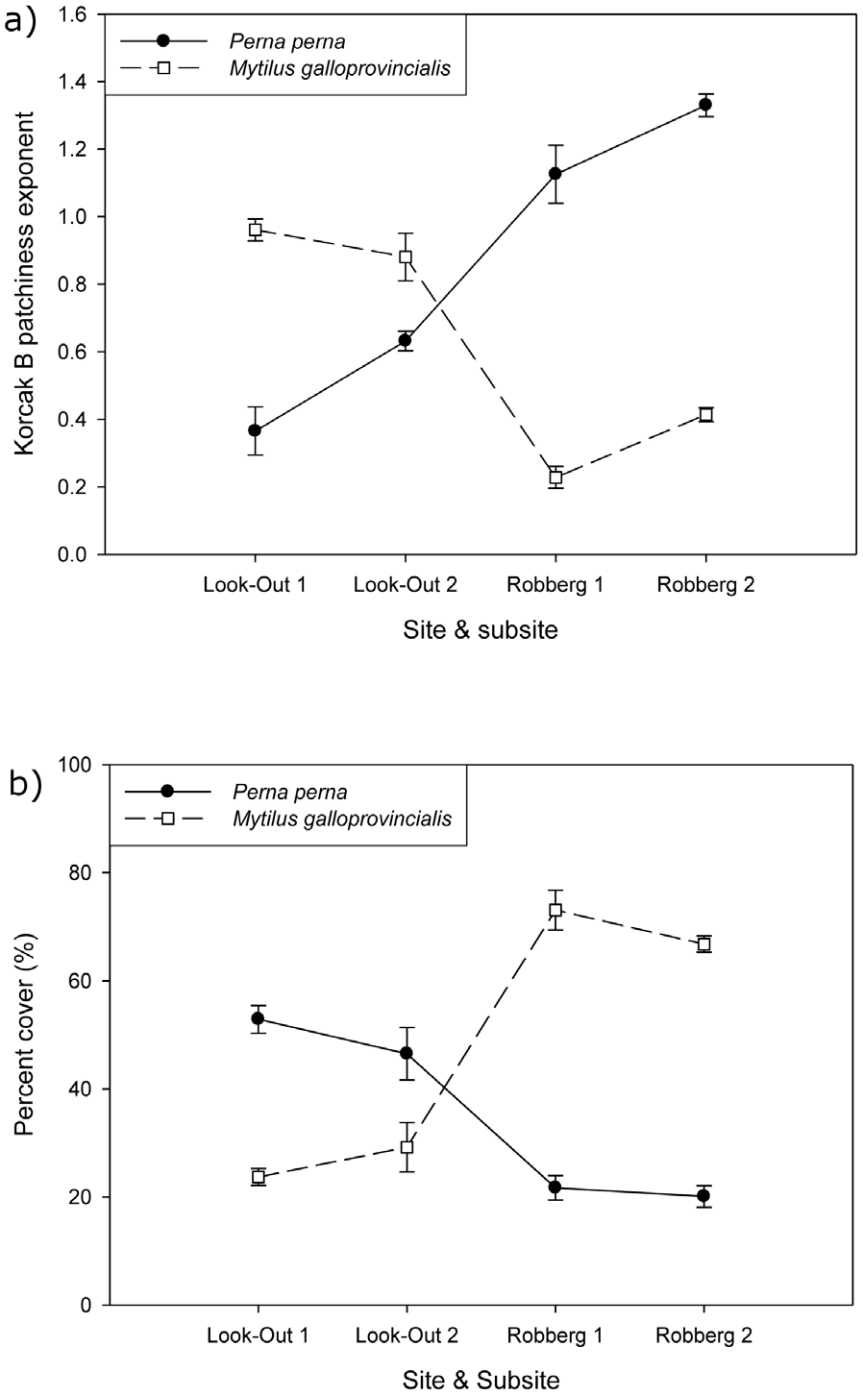
Differences in a) patchiness and b) mussel cover between the sites (Look-Out; Robberg) and sub-sites.

At both sub-sites in Look-out Beach there was significantly higher cover of *P. perna* than *M. galloprovincialis*, while patchiness was significantly lower for *P. perna* (Korcak B estimates closer to 0) than for *M. galloprovincialis* (B closer to 1; Table 1 & 2; Fig. 2; SNK-post hoc test, P<0.05). In contrast, at both sub-sites in Robberg, there was significantly higher cover of *M. galloprovincialis* than *P. perna*, with patchiness significantly lower for *M. galloprovincialis* than for *P. perna* at this site (see Table 1, 2; Fig. 2; SNK-post hoc test, P<0.05). The two 3-factor nested ANOVAs of Korcak B index and percent cover of mussels respectively showed slightly heterogeneous variances (Cochran's test: SQRT (Korcak B+1) transformed data, C = 0.3016, C_crit_ = 0.2926, p<0.05; percent cover untransformed data, C = 0.3004, C_crit_ = 0.2926, p<0.05). However, slightly heterogeneous variances are not a problem when samples are balanced and when the numbers of different treatment groups and samples are relatively large, usually more than 5 treatments and n larger than 6, as ANOVA is robust enough for this [45], [47]. In the present study the numbers of treatment groups and samples were 8 and 10 respectively.

**Table 1:** Three-factor nested ANOVA (Sub-site nested in Site; Species a fixed factor) of Korcak B patchiness exponent of mussels in the mid zone.

Source of variation	df	MS	F	p	Error term
Species	1	0.1600	0.12	0.7839	Spec.×Site
Site	1	0.0055	0.15	0.7377	Subsite(Site)
Subsite(Site)	2	0.0373	9.32	0.0003	Residual
Spec.×Site	1	1.2823	49.73	0.0195	Spec.×Subs.(Site)
Spec.×Subs.(Site)	2	0.0258	6.43	0.0027	Residual
Residual	72	0.0040			

Analysis done of square root transformed (SQRT (B+1)) data, which made variances more homogeneous, although still slightly heterogeneous.

**Table 2:** Three-factor nested ANOVA (Sub-site nested in Site; Species a fixed factor) of percent cover of mussels in the mid zone.

Source of variation	df	MS	F	p	Error term
Species	1	3328.20	0.13	0.7818	Spec.×Site
Site	1	1080.45	13.67	0.0660	Subsite(Site)
Subsite(Site)	2	79.02	0.81	0.4499	Residual
Spec.×Site	1	26136.45	127.73	0.0077	Spec.×Subs.(Site)
Spec.×Subs.(Site)	2	204.62	2.09	0.1310	Residual
Residual	72	97.84			

Analysis of non-transformed data as transformed data did not make variances more homogeneous.

**Figure 3:**
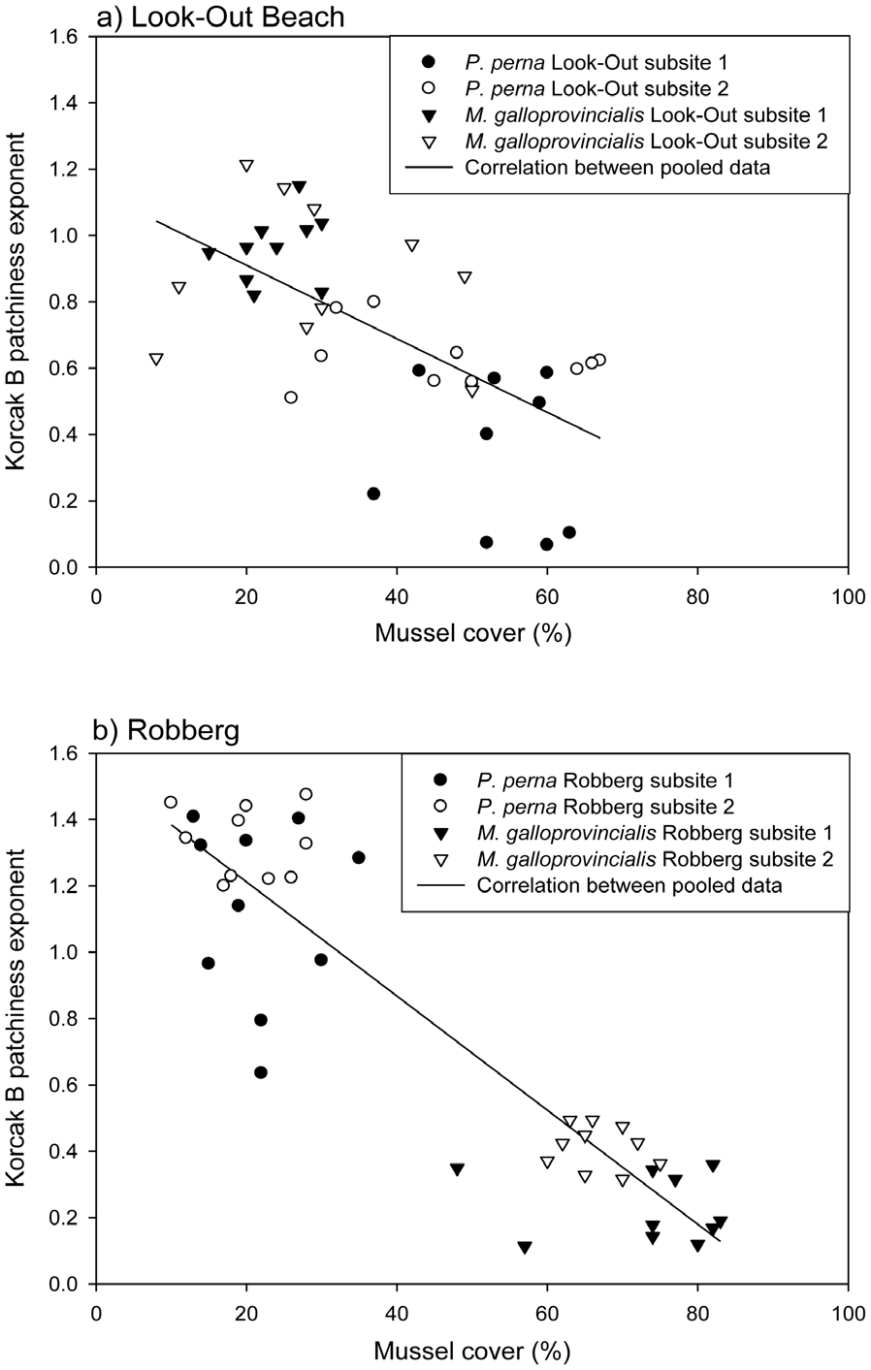
Linear regression between patchiness index and mussel cover at a) Look-Out and b) Robberg.

Thus, at each site the species with the highest cover had the lowest B estimates (patchiness low), indicating that it showed fewer, larger patches, while the species with the lowest cover had high B estimates (patchiness high) indicating that its distribution consisted of more, smaller patches. However, the correspondence between percent cover and patchiness of mussels between sites was not maintained within sites for either species, since there was no significant negative correlation between percent cover and Korcak B estimates of each species at each site. (such a correlation would be very hard to detect since the range of observed cover was very narrow within subsites), although correlations were significant if species and/or sites were pooled (Fig. 3; both species combined at: Look-Out 1: t = −5.91, p = 0.00001, r = −0.81; Look-Out 2: t = −1.96, p = 0.06, r = −0.42; Robberg 1: t = −7.63, p = 0.0000001, r = −0.87; Robberg 2: t = −15.48, p = 0.0000001, r = −0.96; Look-Out 1+2: t = −5.03, p = 0.00001, r = −0.63; Robberg 1+2: t = −13.42, p = 0.0000001, r = −0.91). All Korcak B estimates (i.e. the slope of the log-log regression between N and i) showed r^2^-values ranging between 0.64 and 0.99 (mean = 0.89, SD = 0.08) indicating that log-log linear regressions between N and i (the number of patches with i individuals in) were very good.

## Discussion

Monospecific populations of soft-bottom blue mussels are known to show strong self-organisation in both the laboratory and the field [7]. Here we show that both non-random and random distribution of two mussel species (i.e. conspecific or mixed aggregation) can occur at scales that are small relative to body size under field conditions on rocky shores, and that patchiness is related to the numerical dominance of each species. This is important, as it is linked to the long-term co-existence of two ecologically similar species in situations where at least one resource (space) is limiting. In this system co-existence could be explained by the balance between facilitation and competition between the two species. If populations of these species are shaped primarily by interspecific competition, then we would expect conspecific aggregation, while facilitation would promote mixed (random) aggregation. In the event we observed both mixed and conspecific aggregation, which presumably reflects that in the mid-mussel zone both competition and facilitation occur [27].

There is overlapping habitat use by our two study species, i.e. they co-exist in the mid-mussel zone with each being excluded from one part of the shore by environmental stress or a combination of competition and environmental stress. *M. galloprovincialis* has weaker byssal attachment [48] and is excluded from the low shore by its susceptibility to wave action as well as competitive exclusion by *P. perna* [24]. *P.perna* is excluded from the upper mussel zone by recruitment failure [25] and its vulnerability to desiccation [27]. Thus *P.perna* is excluded from the high mussel zone and *M. galloprovincialis* from the low mussel zone largely by pre- and post-recruitment processes respectively [49].

Here, we showed that the two species can exhibit both conspecific and mixed aggregation (i.e. context-dependent aggregation) in the mid-mussel zone. Conspecific aggregation can be viewed as a consequence of interspecific competition (individuals are eliminated by competitors) or as active aggregation that could be established at different ontogenetic stages. Mussels have external fertilisation with dispersive planktonic larvae, and there is evidence for differences between the two mussels in spawning seasonality [50]. When coupled with seasonality in the ocean currents that disperse larvae, this can affect dispersal and population connectivity [51], and could result in conspecific aggregation as conspecific larvae will tend to arrive on the shore together. Secondly, larvae of these species show changes in their responsiveness to the presence of other recruits as they age. Field experiments indicate that larvae are attracted to biofilm as they settle out of the plankton, while older recruits (late plantigrades) are attracted to the combination of biofilm and other recruits [52]. This indicates that settler behaviour may be flexible so that conspecific attraction at the settlement stage is a possibility. Recruits (i.e. individuals that have settled and undergone metamorphosis) tend to move and clump together faster than adults (Porri et al. unpubl. data), while among adults, *M. galloprovincialis* is significantly more mobile than *P. perna* [53]. In the laboratory, medium-large sized (4–10 mm) recruits of *P. perna* show preferential movement towards conspecific adults (unpubl. data), but we have no information on how individual movement could allow conspecific aggregation under field conditions. A possibility unrelated to mussel behaviour is predator preference for one of the two species, although there is evidence that top-down control can eliminate rather than promote spatial structure [54]. Predators have significant effects on recruits of these species under experimental field conditions [55], but there is no evidence that they exhibit preferences between the two species at such sizes. The situation is different for adults as oystercatchers (*Haematopus moquini*) selectively feed their chicks with *M. galloprovincialis* where both mussel species are available [56], but it seems unlikely that this could produce the observed patterns. Although secondary settlers of mussels show attraction to other mussel recruits [52], we have no evidence that this attraction is species-specific. Overall it seems likely that conspecific aggregation results from a combination of such attraction plus synchronised settlement of monospecific clouds of larvae (although more extensive studies need to confirm this pattern).

Analyses of patchiness in the mid zone revealed that patchiness and numerical dominance can be linked depending on scale. The two are related among sites and species, so that the numerically dominant species at a given site had lower patchiness, but this effect did not occur within species and site (i.e. no significant correlation between percent cover and Korcak B estimates of each species at each site). This may simply reflect the probability of coalescence of small patches into fewer larger patches as abundance increases. In a similar way the probability of mussel removal by storms in the mid zone at the same sites is related to the numerical dominance of each species [11] conforming to the compensatory mortality model described by Connell [57]. We speculate that the negative relationship between patchiness and percent mussel cover would differ depending on the balance between competition and facilitation. With facilitation alone, there would be no competitive exclusion and a weak effect of cover on B (i.e. no significant relationship), while, with no facilitation, competition would result in exclusion of the weaker competitor and a strong negative effect of total mussel cover on B for that species as its population becomes more fragmented by more intense competition. Indeed, there is a trend (Fig. 3) that the slope of the curve at Robberg (total mussel cover ca 85–100%) is steeper than at Look-Out Beach where total mussel cover was lower (ca 70–85%), and competition for space presumably weaker.

There are a number of interactions between the two species that can be considered as facilitation, as defined by Bruno et al. [32]. For example, *P. perna* is extremely slow to recolonise free primary space after experimental removal [58] and its settlement is facilitated by the presence of *M. galloprovincialis*, which recolonises free space 2–3 times faster following disturbance in the mid zone [11]. Furthermore, field experiments have shown that in the mid- mussel zone *P. perna* is weakly facilitated by *M. galloprovincialis* as adult *P. perna* survive better when mixed with *M. galloprovincialis*, indicating that *P. perna* is to some extent also protected from desiccation by the physical matrix provided by the more desiccation-tolerant *M. galloprovincialis* [27]. This is presumably related to the greater susceptibility of *P. perna* to hypoxia; during aerial exposure it is obliged to gape the shell to allow oxygen uptake, making it more vulnerable to desiccation [26].

Similarly, on the low shore the survival of *M. galloprovincialis* is initially facilitated by *P. perna*. *M. galloprovincialis* in experimental monospecific patches on wave-exposed shores show much higher mortality than when mixed with *P. perna*, although it is ultimately ousted by interference competition for space [24]. Thus each species is partially facilitated by the other in zones where it is physically challenged through amelioration of abiotic stress, though this does not prevent partial habitat segregation. *M. galloprovincialis* appears to have two competitive advantages in the mid zone: firstly, faster recolonisation of free space [11], and secondly *P.perna* exhibits intraspecific competition in this zone [27]. However, *M. galloprovincialis* also ameliorates desiccation of *P.perna*, allowing it to survive there, so that the patchy pattern observed in the mid zone may be explained by a combination of different factors.

**Figure 4:**
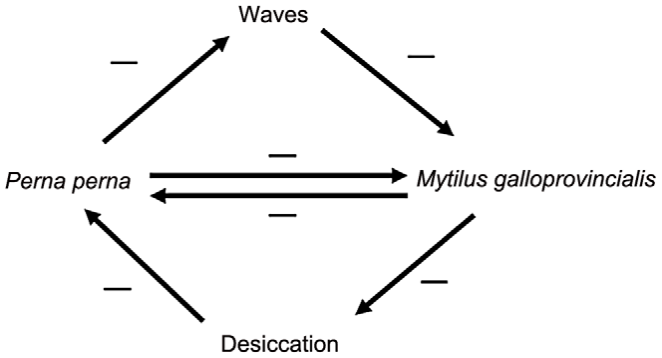
Summary of suggested explanations of how two negative effects interact to produce a positive effect. The amelioration of abiotic stresses by the more tolerant species constitutes an indirect positive effect (two negative effects making a positive one) on the less tolerant competitor. In the case of wave action, this is an indirect effect of *P. perna* on *M. galloprovincialis*. In the case of desiccation stress, the reverse is true. Direct interactions between the two are negative.

The observation that co-existence, even at small spatial scales (cm-m scales), is possible between two species that compete for at least one and probably two resources (space and food) challenges existing theory, especially as one is indigenous and the other invasive. Importantly, this is possible because the two species both compete and facilitate each other's survival [27]. This occurs because both species are ecosystem engineers that modify the physical environment, ameliorating conditions for their competitor, and is in contrast to Branch's [10] suggestion that competitive exclusion on rocky shores is likely when two species compete for space. Co-existence at these small spatial scales is possible through the balance between direct (competitive) negative interactions and indirect (facilitative) interactions that are positive in sum, because they combine two negative effects (Fig. 4).
